# MiR-155 promotes epithelial-mesenchymal transition in hepatocellular carcinoma cells through the activation of PI3K/SGK3/β-catenin signaling pathways

**DOI:** 10.18632/oncotarget.11800

**Published:** 2016-09-01

**Authors:** Xin Kong, Fengchao Liu, Jian Gao

**Affiliations:** ^1^ Department of Gastroenterology, Second Affiliated Hospital, Chongqing Medical University, Chongqing, China

**Keywords:** epithelial-mesenchymal transition, SGK3, miR-155, hepatocellular carcinoma, phosphatidylinositol-3-kinase

## Abstract

Oncogenic mutations in PIK3CA, the gene encoding the catalytic subunit of phosphoinositide 3-kinase (PI3K), occur with high frequency in hepatocellular carcinoma (HCC). The protein kinase Akt is considered to be the primary effector of PI3K, but there is evidence to suggest that serum and glucocorticoid kinase 3 (SGK3) acts in an Akt-independent manner downstream of PI3K. In this report, we found that SGK3 promotes epithelial-mesenchymal transition (EMT) and reduces phosphorylation-dependent degradation of β-catenin in HCC cells. We determined that miR-155, previously shown to promote EMT, stimulates the expression of SGK3 by targeting and repressing P85α, thereby removing its inhibitory effect on PI3K-AKT signaling. These findings suggest that miR-155 promotes EMT and metastatic properties in HCC cells through activation of PI3K/SGK3/β-catenin signaling pathways.

## INTRODUCTION

Hepatocellular carcinoma (HCC) is one of the most common liver malignancies and the second most frequent cause of cancer related death [1]. Metastasis is the primary cause of death in cancer patients, and EMT (EMT) is predicted to be necessary for metastatic progression. The complex transcriptional and posttranscriptional regulatory network that controls the EMT process provides an attractive target to disrupt the progression of metastasis.

MicroRNAs (miRNAs) are short, ∼22 nucleotide noncoding RNAs that target specific mRNAs for cleavage or translational repression [2] and play important roles in cancer pathogenesis, acting as either oncogenes or tumor suppressors [3]. Many miRNAs, including miR-155, stimulate HCC development [4]; of these, several are associated with EMT [5]. MicroRNA-155 (miR-155) acts as an oncogene and is up-regulated in several human cancers, including HCC [6] and our prior research demonstrates that miR-155 promotes EMT and cancer stem cell phenotypes [7, 8].

The phosphatidylinositol 3 kinase (PI3K) pathway is one of the most important pathways in cancer metabolism and growth [9]. Arguably the best-understood effector of PI3K is the serine/threonine protein kinase Akt/protein kinase B. A recent study showed that PIK3R1 (p85α), a negative regulator of the phosphatidylinositol 3-kinase (PI3K)–AKT pathway, is a direct target of miR-155 [10]. Our previous results indicated that Akt1 promotes enrichment of cancer stem cell-like cells and chemoresistance in HCC cells [11], but despite numerous studies pointing to Akt as a primary transducer of the PI3K signal, PIK3CA mutant tumors have strikingly low levels of phosphorylated (activated) Akt, indicating that other effectors must link PI3K to tumorigenesis [12]. A recent study suggested that SGK3 promotes breast cancer through an Akt-independent mechanism and promotes cell proliferation and survival in hepatocellular carcinoma [13–15].

**Figure 1:**
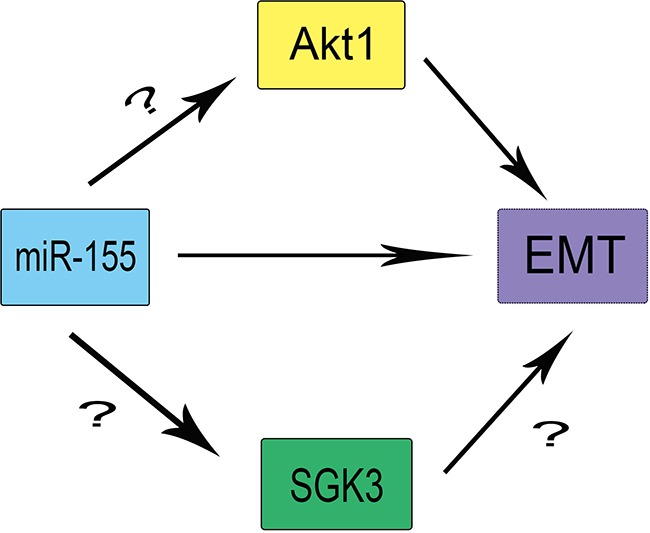
The theoretical roles and regulation of SGK3, miR-155, and Akt1 in EMT

The AGC protein kinase family encompasses three isoforms SGK1, SGK2, and SGK3, which share ∼55% sequence identity with the Akt1-3 catalytic domains [16–18]. Like Akt, SGKs are reportedly involved in the regulation of cell growth, proliferation, survival, and migration [19, 20]. SGK3 has been implicated in the regulation of the interleukin(IL)-3-dependent survival pathway [21], but there is no evidence demonstrating a direct relationship between SGK3 and EMT. Also, whether miR-155 acts through SGK3 or Akt to promote EMT in HCC cells is not clear. Furthermore, the proteins functioning downstream of SGK3 remain unknown (Figure 1). We sought to examine the regulation of SGK3 through miR155 and whether SGK3 plays a significant role in EMT of HCC cells.

## RESULTS

### SGK3 promotes cell migration and invasive potential in HCC cells

**Figure 2:**
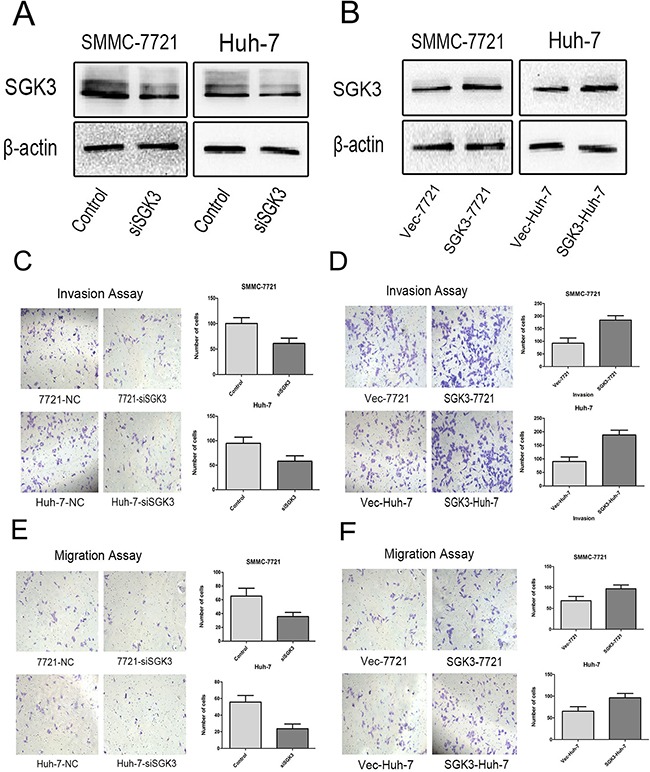
SGK3 promotes cell migration and invasive potential **A.** Depletion of SGK3 in SMMC-7721 and Huh-7 cells. Cells were transfected with siRNAs against AGK3 and controls, and then subjected to Western blotting with an anti-SGK3 antibody. β-actin was used as loading control. **B.** Overexpression of SGK3 in SMMC-7721 and Huh-7 cells. Cells were transfected with empty vector or a vector expressing SGK3, and then subjected to Western blot analysis with an anti-SGK3 antibody. β-actin was used as loading control. **C.** Control and SGK3-depleted SMMC-7721 and Huh-7 cells were subjected to a transwell Matrigel invasion assays **D.** Empty vector and SGK3-overexpressing SMMC-7721 and Huh-7 cells were subjected to a transwell Matrigel invasion assays **E.** Control and SGK3-depleted SMMC-7721 and Huh-7 cells were subjected to a transwell migration assays **F.** Empty vector and SGK3-overexpressing SMMC-7721 and Huh-7 cells were subjected to transwell migration assays. All experiments were performed in triplicate, and the results are shown as the mean ± standard deviation.

Studies suggest that SGK3 has strong oncogenic potential and is amplified and hyperactivated in breast cancer and hepatocellular carcinoma [14, 15]. We hypothesized that SGK3 is potentially oncogenic due to its regulation of EMT in liver cells. We used siRNA to silence the expression of SGK3 in the HCC cell culture lines SMMC-7721 and Huh-7. (Figure 2A). Because EMT increases cell motility and invasiveness [22, 23], we tested the effect of SGK3 depletion on cell migration and invasion ability by performing transwell migration and invasion assays. Silencing the expression of SGK3 decreased invasion ability (P<0.02) and migration ability (P<0.02) in both SMMC-7721 and Huh-7 cells (Figure 2C, 2E)

**Figure 3:**
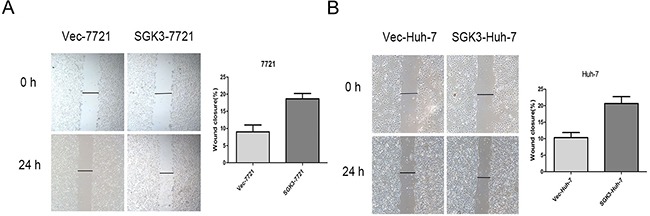
Overexpression of SGK3 promotes wound-healing capacity of HCC cells **A.** A wound-healing assay was performed to determine the migratory capacity of SMMC-7721 cells transfected with empty vector or SGK3-overexpressing lentiviral vector. **B.** A wound-healing assay was performed to determine the migratory capacity of Huh-7 cells transfected with empty vector or SGK3-overexpressing lentiviral vector. All experiments were performed in triplicate, and the results are shown as the mean ± standard deviation.

Lentiviral overexpression of SGK3 in SMMC-7721 and Huh-7 cells (Figure 2B) enhanced their invasion (Figure 2D) (P<0.01 for both) and migration ability (Figure 2F) (P<0.01 and P<0.03). Overexpression of SGK3 increased the wound-healing capacity, and therefore migration ability, of both SMMC-7721 cells (Figure 3A) (P<0.01) and Huh-7 cells (Figure 3B) (P<0.01).

### SGK3 promotes the expression of epithelial and mesenchymal markers

**Figure 4:**
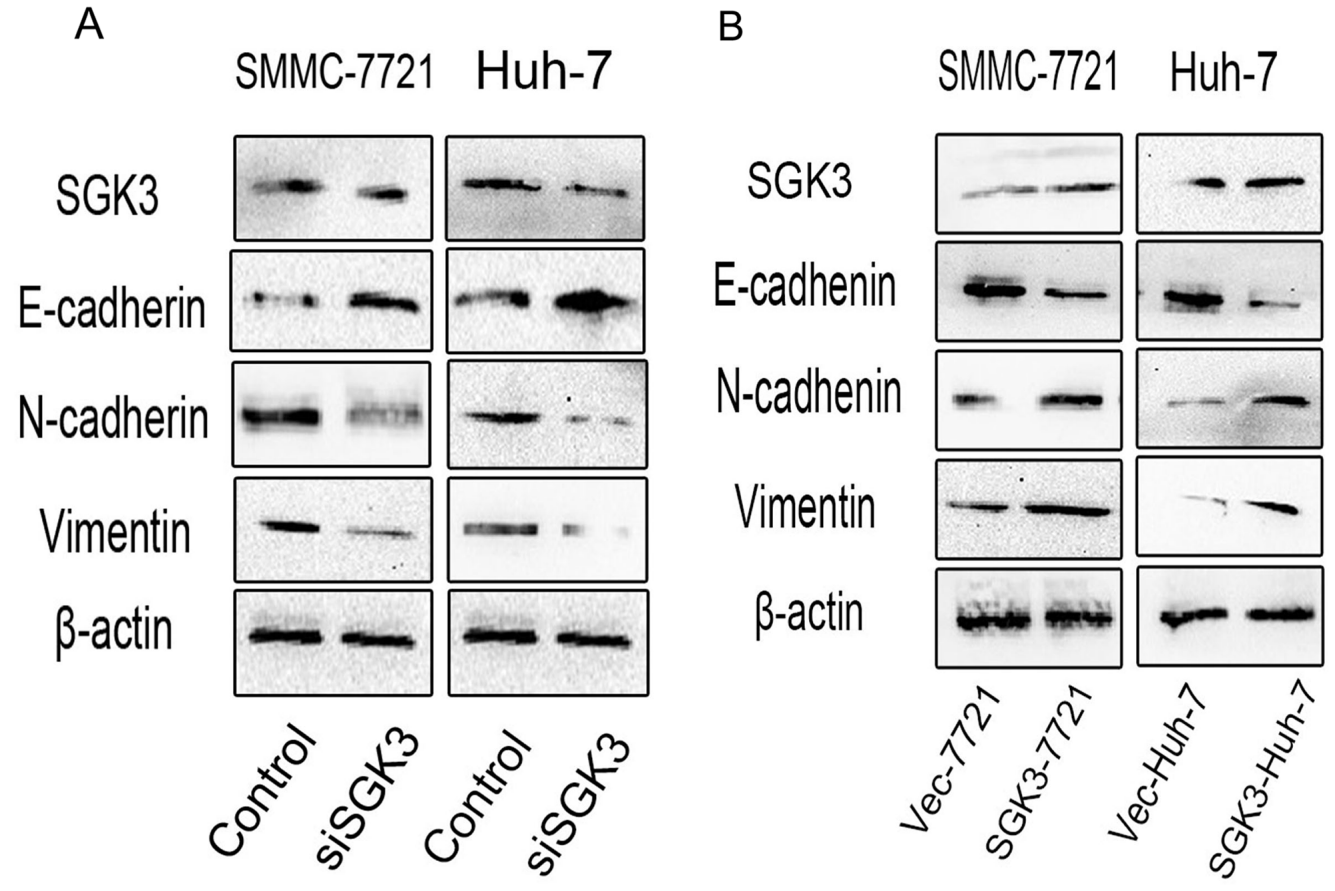
SGK3 regulates the expression of epithelial and mesenchymal markers **A.** Depletion of SGK3 resulted in a gain of E-cadherin expression and a loss of N-cadherin and Vimentin expression in SMMC-7721 and Huh-7 cells. **B.** Overexpression of SGK3 resulted in a loss of E-cadherin expression and a gain of N-cadherin and Vimentin expression in SMMC-7721 and Huh-7 cells. The expression of E-cadherin, N-cadherin and Vimentin was detected by Western blot analysis. β-actin was used as loading control. All experiments were performed in triplicate.

Enhanced cell migration and invasion properties are important consequences of EMT [24]. Morphological and phenotypic EMT-like changes are accompanied by the down-regulation of the epithelial marker E-cadherin and the up-regulation of mesenchymal markers Vimentin and N-cadherin [25, 26]. Depletion of SGK3 in SMMC-7721 and Huh-7 cells led to the up-regulation of E-cadherin and the down-regulation of both N-cadherin and Vimentin (Figure 4A). Overexpression of SGK3 in SMMC-7721 and Huh-7 cells led to the down-regulation of E-cadherin and the up-regulation of both N-cadherin and Vimentin (Figure 4B).

### MiR-155 stimulates EMT through activation of SGK3 by targeting P85α

**Figure 5:**
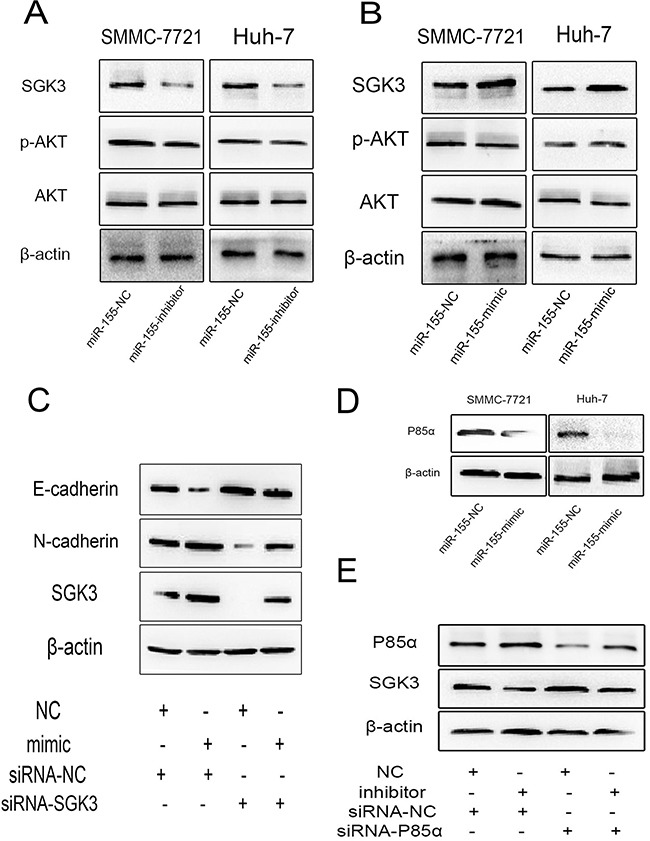
miR-155 stimulates EMT through by targeting P85α to activate SGK3 The expression of SGK3, p-AKT, AKT in SMMC-7721 and Huh-7 cells **A.** treated with miR-155 inhibitors or **B.** overexpressing miR-155 was measured by Western blot analysis. β-actin was used as a loading control. All experiments were performed in triplicate. **C.** The suppression of SGK3 in SMMC-7721 cells transiently overexpressing miR-155 resulted in a rescue of EMT inhibition detected by western blot. **D.** Western blot shows decreased expression of endogenous P85α due to miR-155 overexpression in SMMC-7721 and Huh-7 cells. **E.** The suppression of P85α in SMMC-7721 cells with transiently repressed miR-155 resulted in a rescue of SGK3 suppression detected by western blot.

Our prior research revealed that miR-155 could promote EMT in HCC cells [7]. We hypothesized that miR-155 may exert its effect of EMT through a relationship with SGK3. MiR-155 was transiently down-regulated following transfection of miR-155 inhibitor or NC in both SMMC-7721 and Huh-7 cells. At 48 h post transfection, we detected the expression of SGK3, p-Akt and total Akt by Western blot analysis. Down-regulation of miR-155 decreased the expression of SGK3 (Figure 5A), but had no apparent effect on the level of p-Akt.

Next, we used miR-155 mimics or NC to up-regulate miR-155 in both SMMC-7721 and Huh-7 cells. The expression level of SGK3 was increased in the miR-155 mimics group compared with the NC group in both SMMC-7721 and Huh-7 cells. However, there was no apparent effect on the level of p-Akt (Figure 5B).

To verify whether silencing SGK3 largely reduces the miR-155 induced EMT, a rescue experiment was performed. We transfected SMMC-7721 cells with mimics to miR-155 plus SGK3 siRNA or with the same mimics plus NC, cells co-transfected SGK3 siRNA and miR-155 mimics rescued the inhibition of EMT by SGK3 siRNA (Figure 5C).

Recent studies show that P85α, a negative regulator of the phosphatidylinositol 3-kinase (PI3K)–AKT pathway, is a direct target of miR-155 [10]. As expected, P85α protein expression was significantly down-regulated in miR-155 overexpressing SMMC-7721 and Huh-7 cells compared to miR-NC (Figure 5D). We performed a rescue experiment to verify whether miR-155 promotes the expression of SGK3 by targeting and repressing P85α. Co-transfection with miR-155 inhibitors and P85α siRNA rescued the suppression of SGK3 by miR-155 inhibitors (Figure 5E).

### SGK3 stimulates β-catenin signaling in HCC cells

**Figure 6:**
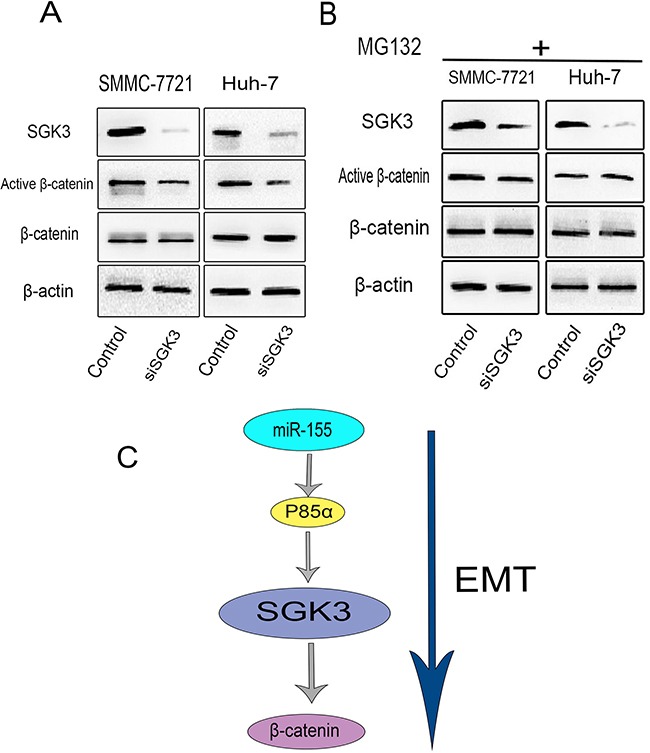
SGK3 regulates β-catenin signaling in HCC cells **A.** The expression of SGK3, active β-catenin and total β-catenin were detected by Western blot analysis in SMMC-7721 and Huh-7 cells with SGK3 knocked down. β-actin was used as a loading control. **B.** Use of the proteasome inhibitor MG132 (10 μM; 8 hours) prevented the degradation of β-catenin induced by SGK3 depletion. All experiments were performed in triplicate. **C.** Schematic illustration of SGK3 promotion of EMT. miR-155 stimulates the expression of SGK3 to promote β-catenin signaling in HCC cells.

Loss of E-cadherin often leads to the up-regulation of β-catenin signaling pathways [27, 28] and increased transcriptional activity of β-catenin is associated with EMT [22, 29, 30]. In the WNT signaling pathway, GSK-3β forms a destruction complex with Axin and adenomatous polyposis coli (APC) for the phosphorylation and subsequent degradation of β-catenin. There is evidence to suggest that SGK3 promotes the phosphorylation of GSK-3β on Ser9 [15]. However, a direct relationship between SGK3 and β-catenin has not been demonstrated. Therefore, we sought to elucidate a potential role for SGK3 in β-catenin signaling. Reduction of SGK3 expression using siRNA in SMMC-7721 and Huh-7 cells markedly reduced the level of active β-catenin (dephosphorylated on Ser37 and Ser41) (Figure 6A). This effect of SGK3 depletion on β-catenin was blocked in the presence of the proteasome inhibitor MG132, suggesting that the degradation of β-catenin is mediated by the proteasome (Figure 6B). Taken together, these data indicate a novel role of SGK3 in the β-catenin pathway in HCC cells (Figure 6C).

## DISCUSSION

The SGK family exhibits structural similarity to Akt, arousing interest in their functions because Akt plays important roles in cell survival, proliferation, and metabolism. We sought to understand the role of SGK3 in regulation of EMT. Down-regulation of SGK3 decreased the migration and invasive potential of SMMC-7721 and Huh-7 cells and resulted in up-regulation of the epithelial marker E-cadherin and down-regulation of the mesenchymal markers N-cadherin and Vimentin. Overexpresssion of SGK3 had the inverse effect, increasing the wound-healing capacity of HCC cells.

MicroRNA-155 (miR-155) acts as an oncogene and is overexpressed in several types of human cancers, including hepatocellular carcinoma [6]. It should be noted that miR-155 has many cancer-related targets, such as P85α and DMTF1 [10, 31]. We now propose that miR-155 regulates SGK3 by targeting and repressing P85α, which in turn stimulates EMT in HCC cells. This is supported by the fact that miR-155 inhibitors decreased the expression of SGK3 but had no effect on the levels of Akt. miR-155 mimics increased SGK3 levels and, to a lesser extent, Akt levels. The rescue experiment proves that the effect of miR-155 on EMT could be blocked by SGK3 siRNA in SMMC-7721 cells. In SMMC-7721 and Huh-7 cells, up-regulation of miR-155 decreased the expression of P85α. In our rescue experiment, P85α siRNA rescued the suppression of SGK3 by miR-155 inhibitors. It should be noticed that miR-155 could directly act on SGK3 to modulate the progression of neuropathic pain [32]. We hypothesize that miR-155 may have different effects in different types of cells.

During tumorigenesis, EMT is associated with aberrant activation of the canonical Wnt pathway, which inactivates GSK-3β and stabilizes β-catenin and snail, respectively [33, 34]. SGK3 enhances the phosphorylation of GSK-3β on Thr9 (inactivated) to inhibit GSK-3β- mediated degradation of β-catenin [35]. We therefore hypothesized that SGK3 also has an effect on the regulation of β-catenin. Our results show that down-regulating SGK3 decreases the level of active β-catenin (dephosphorylated on Ser37 and Ser41). Use of the proteasome inhibitor MG132 blocked SGK3′s effect on β-catenin. Taken together, this suggests that SGK3 can stabilize β-catenin by inhibiting its proteosomal degradation.

In conclusion, our data suggests that miR-155 promotes EMT in HCC cells through the PI3K/SGK3 signaling pathway. Furthermore, SGK3 promotes the dephosphorylation of β-catenin to prevent its degradation in HCC cells. Future in vivo experiments will be performed to study the role of SGK3 in hepatocellular cancer stem cells to characterize the pathogenesis of HCC.

## MATERIALS AND METHODS

### Cell lines and culture

Human HCC cell lines Huh-7 and SMMC-7721 were purchased from the cell bank of the Chinese Academy of Sciences, Shanghai, China, in October 2014. All cell lines have been authenticated using DNA-Fingerprinting in the cell bank and confirmed to be mycoplasma-negative using Hoechst staining. We did not find other microorganisms in these new cell lines. Cells used for experiments were passaged for fewer than five generations. All cell lines were maintained in Dulbecco's modified Eagle medium (DMEM; Gibco) supplemented with 10% fetal bovine serum (FBS; Gibco), 100 units/ml penicillin, and 100 ug/ml streptomycin in a 37°C incubator with 5% CO_2_.

### Cell transfection

MiR-155 mimics, inhibitors, SGK3 siRNA, and negative controls (NC) were designed and synthesized by GenePharma (Shanghai, China). Sequences used for SGK3 siRNA, p85α siRNA, and NC were 5′-GCAUUGGGUUACAUUTT-3′,5′-AACAG CAACUGGCUAUGGCdTdT-3′and 5′-UGACCUCAA CUACAUGGUUTT-3′, respectively. Sequences used for the miR-155 mimics and the corresponding control were 5′-UUAAUGCUAAUCGUGAUAGGGGU-3′ and 5′-UUCUCCGAACGUGUCACGUTT-3′, respectively. Sequences used for the miR-155 inhibitors and the corresponding control were 5′-ACCCCUA UCACGAUUAGCAUUAA-3′ and 5′-UUCUCCGAAC GUGUCACGUTT-3′, respectively. The cells were transfected using siRNA-MateTM (GenePharma) according to the manufacturer's protocol. Briefly, the cells were seeded at 2-3×105 cells per well in 6 well cell culture plates and grown to 30%-50% confluence. Transfection complexes were prepared according to the manufacturer's instructions and were added directly to the cells. The SGK3 inhibitor, miR-155 mimics, and negative controls were used at a final concentration of 50 nM, and the miR-155 inhibitors, siRNA, and negative controls were used at a final concentration of 100 nM. Western blotting was used to determine the level of protein expression after incubation with siRNA for 48 h.

### Establishment of the SGK3 stable expression cell line

To establish stable transduction, we purchased a high-expressing lentiviral vector from GenePharma containing the SGK3 or control primary transcripts (Shanghai, China). Transfection was performed using Polybrene (GenePharma) according to the manufacturer's instructions. Briefly, the cells were seeded at 1×10^5^ cells per well in a 24 well cell culture plate and grown to 20%-40% confluence. Next, the cells were transfected at a multiplicity of infection of 40 in the presence of 8 μg/ml Polybrene. After 72-96 h, protein expression was verified by fluorescence microscopy.

### Transwell migration assay and Matrigel invasion assay

For the Transwell migration assay, 1×10^4^ cells were seeded per well; 5×10^4^ cells/well were seeded for the invasion assay. Cells were resuspended in 200 μl of serum-free DMEM and seeded in the upper chamber of a 24-well Transwell® plate with an 8.0-μm pore polycarbonate membrane insert (Corning). A total of 700 μl of culture medium containing 10% fetal bovine serum was added to the lower chamber to create a gradient. The inserts was maintained at 37°C in 5% CO_2_ for 24 h. Next, the non-invading cells in the upper chamber were gently removed with a cotton swab. The cells migrating through the polycarbonate membrane were fixed with 4% paraformaldehyde for 20 minutes and stained with 0.1% crystal violet for 20 minutes. The cells were counted under a microscope in five randomized fields. For the cell invasion assay, the steps were the same as the migration assay, except that the polycarbonate membrane was coated with Matrigel (BD Biosciences), and the inserts were maintained at 37°C in 5% CO_2_ for 48 h.

### Wound healing assays

After the cells were grown to approximately 100% confluence in a 6 well cell culture plate, wound healing assays were performed with a 200-μl sterile pipette tip to make a scratch through the confluent monolayer. The old culture medium was then replaced with serum-free medium. The cells were maintained at 37°C in 5% CO_2_. Five marked fields were observed at 0 and 24 h to assess the rate of gap closure.

### Protein extraction and Western blotting

Total protein was extracted from the cells using RIPA lysis buffer (CWBIO), and the protein concentration was determined using a BCA Kit (Pierce). The proteins were separated by sodium dodecyl sulfate–polyacrylamide gel electrophoresis and transferred to polyvinylidene difluoride membranes (Millipore). The membranes were incubated overnight at 4°C with primary antibodies after blocking with 5% skim milk. The membranes were subsequently washed using Tris-buffered saline with 0.1% Tween and incubated with horseradish peroxidase-linked secondary antibody for 1 h. The blots were visualized using enhanced chemiluminescence reagent (Engreen). The following antibodies were used: Rabbit anti-E-cadherin (ab40772; abcam), Rabbit anti-β-actin (YT0099, Immunoway), Rabbit anti-vimentin (ab92547, abcam), Rabbit anti-N-cadherin (ab76011;abcam), Rabbit anti-Akt1 (ab32505), Rabbit anti-Akt1 (phosphoS473) (ab81283), Rabbit anti-SGK3 (#5642;CST), Rabbit anti-β-catenin(#8480, CST), Rabbit anti-non-phospho (Active) β-Catenin (Ser33/37/Thr41) (#8814, CST), Rabbit anti-AKT1 (S473)(ab81283), Mouse anti- p85α(#13666;CST).

### Statistical analysis

SPSS 22 software was used for statistical analysis. All data were acquired from at least three independent experiments and are presented as the mean ± SD. Student's t-test was used for comparisons between two independent groups. P<0.05 was considered to be statistically significant.
